# Mechanical Behavior of Bi-Layer and Dispersion Coatings Composed of Several Nanostructures on Ti13Nb13Zr Alloy

**DOI:** 10.3390/ma14112905

**Published:** 2021-05-28

**Authors:** Dorota Rogala-Wielgus, Beata Majkowska-Marzec, Andrzej Zieliński, Michał Bartmański, Bartosz Bartosewicz

**Affiliations:** 1 Division of Biomaterials Technology, Faculty of Mechanical Engineering and Shipbuilding, Gdansk University of Technology, 11 Narutowicza str., 80-233 Gdańsk, Poland; beata.majkowska@pg.edu.pl (B.M.-M.); andrzej.zielinski@pg.edu.pl (A.Z.); michal.bartmanski@pg.edu.pl (M.B.); 2 Institute of Optoelectronics, Military University of Technology, gen. S. Kaliskiego 2, 00-908 Warsaw, Poland; bartosz.bartosewicz@wat.edu.pl

**Keywords:** coatings, composites, electrophoretic deposition, carbon nanotubes, nanocopper, titanium oxide, nanoindentation, hardness, Young’s modulus, titanium alloy

## Abstract

Titanium implants are commonly used because of several advantages, but their surface modification is necessary to enhance bioactivity. Recently, their surface coatings were developed to induce local antibacterial properties. The aim of this research was to investigate and compare mechanical properties of three coatings: multi-wall carbon nanotubes (MWCNTs), bi-layer composed of an inner MWCNTs layer and an outer TiO_2_ layer, and dispersion coatings comprised of simultaneously deposited MWCNTs and nanoCu, each electrophoretically deposited on the Ti13Nb13Zr alloy. Optical microscopy, scanning electron microscopy, X-ray electron diffraction spectroscopy, and nanoindentation technique were applied to study topography, chemical composition, hardness, plastic and elastic properties. The results demonstrate that the addition of nanocopper or titanium dioxide to MWCNTs coating increases hardness, lowers Young’s modulus, improves plastic and elastic properties, wear resistance under deflection, and plastic deformation resistance. The results can be attributed to different properties, structure and geometry of applied particles, various deposition techniques, and the possible appearance of porous structures. These innovative coatings of simultaneously high strength and elasticity are promising to apply for deposition on long-term titanium implants.

## 1. Introduction

Carbon thin coatings continue to attract attention, fueled by their potential for industrial and commercial applications. Carbon nanotubes (CNTs) demonstrate unique properties, such as high aspect ratio, enhanced tensile (tensile strength up to 30 GPa [1]) and elastic properties (elastic modulus of 1 TPa or more [1]), and excellent electrical and thermal conductivity, resulting from their unique molecular structure and dependent on the synthesis method [2]. Extraordinary high mechanical properties led to extensive research on the use of CNTs as a reinforcing agent to produce composites [3,4,5] and laminar coatings [5] with various materials, such as Al_2_O_3_ [6,7,8], hydroxyapatite (HAp) [4,9,10,11,12,13], titanium [4], titanium dioxide (TiO_2_) [5,8,14,15,16], ultra-high molecular weight polyethylene (UHMWPE) [17,18], nickel [19,20,21,22], aluminum [23,24,25], chromium [26], and copper [22]. 

After the reports on CNTs bioactive properties, their application in the biomedical field increased [1,27]. Sasani et al. [28] used copper decorated multi-wall carbon nanotubes (MWCNTs) to incorporate fluorapatite-titania coating onto a Ti-based implant, which improved composite coating in terms of nanomechanical properties. The toxicity profile and biocompatibility of CNTs were the main concerns as confirmed by numerous reports available on this issue [2,29,30,31,32,33,34,35,36,37]. The size (diameter and length [30,31]) and surface area of the CNTs play an important role in toxicology—the smaller the size structure and increased surface area, the larger probability of the interactions with living cells and translocation into the cells, thus adverse biological effects. Kumar et al. [34]. reported on many other important factors determining the cytotoxicity of CNTs, such as the type of CNTs, the type of functionalization, the purification of the product, and dispersion. These parameters should be controlled to reduce the adverse effects of MWCNTs [31,32]. Benko et al. [29] conducted biocompatibility tests on titanium modified with CNTs using the human osteoblast-like cell line MG63. The result confirmed the non-toxic character of the tested materials and showed improvement in the biocompatibility of the modified titanium substrate. What is more, structural disorder and the presence of functional groups, together with an increased surface area of the material, caused better cell adhesion and growth, than for pure titanium [29]. Portan et al. [33] investigated the human bone cells (osteoblasts) behavior when cultured on biomaterials like polymer, titanium, titania nanotubes (TNTs), and CNTs. In this case, osteoblasts cultured on nanostructured topographies, like TNTs and CNTs, improved the proliferation. However, Li et al. [31] reported on MWCNTs harmful activity in the human immune system as impairment of the phagocytic ability, which finally led to cell apoptosis or necrosis. General assumption is that MWCNTs with larger dimensions (long, rigid, a needle-like shape) show higher bioactivity and the shorter the MWCNTs are, the lower the inflammatory response. Literature shows that MWCNTs with a smaller dimension and smaller agglomerate size or with less metal impurities demonstrate lower cytotoxicity [30]. Another study of Zhao et al. [30] revealed that, at the same mass concentration, the cytotoxicity decreased with an increase in the diameter of MWCNTs. Whereas, smaller diameters of MWCNTs generally displayed stronger toxicity towards bacteria [30]. Thus, as so far reports on suspected toxicity are inconsistent and the use of CNTs against bacteria as promising and easy, to achieve titanium surface modification cannot be excluded.

Titanium dioxide (TiO_2_) appears in four natural polymorphs-TiO_2_(B), brookite, anatase, and rutile. Most widely studied are the anatase and rutile crystalline polymorphs, but there is also an amorphous form of TiO_2_ [38]. TiO_2_ is a biocompatible ceramic, which can be used to achieve the antibacterial effect, corrosion resistance, and high biocompatibility in biomedical appliances [5]. For this reason, titanium dioxide is a candidate material for developing protective coatings on metallic substrates [39]. What is more, TiO_2_ is widely used in many different areas, such as gas sensors, and for air purification or dye-sensitized solar cell applications [15]. TiO_2_ has some prominent properties, it is photocatalytically active, has a self-cleaning ability on surfaces, is low-toxic [40]. Ikono et al. [40] carried out cytotoxicity analysis for chitosan-50% TiO_2_ nanoparticles sponges which proved the material to be biocompatible; the cells could proliferate well with a trend of increasing cell number with TiO_2_ concentration. This suggests that such a huge percentage of TiO_2_ nanoparticles in the material could be used for bone tissue engineering. Hsiao et al. [41] also checked TiO_2_ nanoparticles in terms of cytotoxicity which showed that not only the size, surface area, and shape, but also the phase of the nanoparticles played an important role. The cytotoxicity of the TiO_2_ nanoparticles increased in the following order: amorphous > anatase > anatase/rutile. Uboldi et al. [42] showed that rutile induced toxic effects in Balb/3T3 mouse fibroblasts, whereas it was dependent on the particle size. Additionally, Louro [32] claimed that widely used TiO_2_ (as a pigment in paints, varnishes, and plastics, as an additive to food—E171, as UV-filter in cosmetic products), especially the rutile, presented the most genotoxic effects. The literature showed also the induction of micronuclei by anatase TiO_2_ and negative results for rutile when testing genotoxicity using human cell lines [42].

CNTs have a high surface area, which gives them the possibility to build a mesoporous structure, thus they are excellent carrier substrates for TiO_2_ nanoparticles. The combination of CNTs and TiO_2_ nanoparticles can lead to achieving relevant properties for a variety of functional applications. There are some reports on preparing CNTs/TiO_2_ nanoparticle coatings. Boccaccini et al. [43] discussed the mechanism of electrophoretic deposition (EPD) of CNT/TiO_2_ nanoparticle composites and laminates. The subject of the investigation was a four-layer laminate coating of CNTs and TiO_2_, which showed that non-sintered ceramic coating could be reinforced by the addition of CNTs, which provided a crack deflection and delamination path. Jarernboon et al. [15] used CNTs to protect TiO_2_ coating against microcracks. The TiO_2_ coatings deposited using the EPD process showed that the number and length of the microcracks increased with the TiO_2_ layer thickness. The addition of multiwalled carbon nanotubes (MWCNTs) minimized the crack problem because hydroxyl groups and carboxylic groups from CNTs bind to TiO_2_ particles. As a result, microcrack formation was diminished. The reinforcement effect of CNTs was also seen when the CNTs coating was synthesized by air plasma spraying [43]. The CNTs were resistant to damage at increasing temperatures during the spraying process, and the pores of such coatings and their roughness demonstrated to decrease with an increasing percentage of CNTs in the coating microstructure [44]. Jambagi et al. [8] tested plasma-sprayed CNTs/TiO_2_ and CNTs/alumina (Al_2_O_3_) coatings, noticing an improvement of scratch resistance of the listed ceramic coatings. Wang et al. [14] verified the CNTs influence on the friction coefficient of the CNTs/TiO_2_ nanocomposite coating using a ball-on-disk tribometer under dry-sliding conditions. The comparison between nanocomposite and TiO_2_ showed a moderate decrease in friction coefficient and a significant reduction (~93.6%) in wear volume. There were also reports about hydroxyapatite (HAp) coating with titanium and MWCNTs addition (HAp-Ti(20 wt.%)-MWCNTs (1 wt.%) [4] deposited using the EPD method, which showed relevant improvement in hardness and adhesion strength. Moreover, the biological tests revealed an incredible improvement in the apatite and bone cell growth on the nanocomposite coating compared to pure HAp coating.

Copper is an element with proved antibacterial efficiency attributed to its influence on cellular permeability, resulting in malfunction and death of bacteria [45]. Such effects against *S. aureus* and *E. coli* were shown by Hidalgo-Robatto et al. [46] for the nanocopper—HAp coating. The antibacterial activity was observed against *E. coli*, *S. aureus*, and *C. albicans* by Shanmugam and Gopal [47] for Cu substituted HAp coating. For bone phosphate an addition of copper ions eliminated *E. coli*, *P. aeruginosa*, and *S. enteritidis* bacteria [48]. Copper is tolerated by human body, but nanocopper is suspected to destroy the liver and kidneys [49]. The safe content of copper in coatings can be estimated as 2 wt.% as concerns the compatibility [46]. However, as demonstrated by Radovanovic et al. [50] high antibacterial functions can be obtained at low cytotoxicity of coatings. As such, no harmful content of Cu was assessed as of 2 wt.pct in a coating [46]. Moreover, there are several reports of introducing antibacterial properties together with increasing bioactivity and biocompatibility for Cu-incorporated HAp [51], Cu-doped polymer–ceramic coating [52], and Cu-implemented anodic coatings [53].

The most commonly used processing method for the production of ceramic and ceramic-based composite coatings is EPD, which is characterized by many advantages including, low cost, simplicity, low coating time and temperature, control of deposit thickness, uniformity of deposits, the ability to coat the complicated shapes, and microstructural homogeneity [15,54,55,56]. The EPD method has proved to be effective in a relatively simple way to obtain layers from nanometric particles, whose adhesiveness to the substrate may be higher than those containing micrometric size particles [57]. In order to obtain a homogenous deposition, the suspension used in the process has to be well-dispersed and stable [5]. EPD is fundamentally combined with two processes, electrophoresis and deposition. In the first step, material (charged particles) suspended in a liquid is forced to migrate towards an electrode under an applied electric field. In the second step, the particles coagulate at the electrode and form a coherent deposit [4,5,55]. There are numerous publications of CNTs coatings and their hybrids obtained using the EPD process, like [4,5,15,22,54,55,56,58,59,60,61,62,63].

Due to excellent physical (low density, the strength of ~500 MPa and relatively low Young’s modulus close to the value of cortical bone [64]) and chemical properties, titanium and its alloys are applied in medicine, especially in the field of tissue engineering [65]. For long-term bearing implants, mostly Ti6Al4V or Ti6Al7Nb alloys are used. There are also studies about the Ti13Nb13Zr alloy, which show the relatively low Young modulus and its non-toxic potential on the human body (the elements Ti, Nb, Zr are non-toxic) [64,66].

So far, research has shown that surface coatings are numerous as well as their properties, and different determinants for optimization of surface treatment can be chosen. In this research, we have focused only on the creation of two composite coatings which would possess greater hardness at simultaneously high elasticity than the present coatings to prevent possible damage by stresses imposed during surgery and use of an implant. To achieve this objective, we have decided to apply nanoparticles of carbon (carbon nanotubes with high surface/volume ratio), ceramic nanooxides (nanotitania) and nanocopper, in different combinations. The nanoparticles are well-known to have high mechanical properties. We have also assumed that electrophoretically deposited coatings would possess a certain porosity, resulting in lowering Young’s modulus and enlarging the elastic region. Therefore, we have prepared composite coatings as bi-layer (sandwich) deposits obtained by two consecutive processes, and the dispersion coating, formed in a one-stage process.

## 2. Materials and Methods

### 2.1. Surface Preparation

The Ti13Nb13Zr alloy (Xi’an SATE Metal Materials Development Co., Ltd., Xi’an, China) of the composition shown in Table 1 was used as substrate. Specimens of 40 mm in diameter and 4 mm thick slices were cut from the rod and divided into quarters using a precision cutter (Brillant 220, ATM GmbH, Mammelzen, Germany). The surface was ground using abrasive paper SiC up to grit # 800 on a metallographic grinding machine (Saphir 330, ATM GmbH, Mammelzen, Germany). Then, specimens were rinsed with acetone for analysis (Chempur, Piekary Śląskie, Poland), distilled water, dried in the air, picked in 5% hydrofluoric acid (Chempur, Piekary Śląskie, Poland) for 30 s to remove oxide layers from the surface and finally rinsed with distilled water.

The multi-wall carbon nanotubes (MWCNTs) had an outer diameter 10–15 nm, inner diameter 2–6 nm, length 1–10 μm, number of walls 3–15 (3D-Nano, Krakow, Poland). Nanocopper (nanoCu) had a mean grain size distribution of 80 nm (Hongwu International Group Ltd., Guangzhou, Guangdong, China). Titanium dioxide (TiO_2_), rutile structure, (3D-Nano, Krakow, Poland) possessed an extremely small grain size in the range of 1 to 2 nm.

### 2.2. Preparation of MWCNTs/TiO_2_ Bi-Layer and MWCNTs_Cu Dispersion Coatings

The electrophoretic deposition (EPD) method was used to prepare coatings, of which parameters of synthesis are shown in Table 2. The Ti13Nb13Zr substrate was used as an anode and stainless steel as a counter electrode. The electrodes were placed parallel to each other within a distance of 5 mm and connected to the DC power source (MCP/SPN110-01C, Shanghai MCP Corp., Shanghai, China).

The EPD process was conducted at parameters selected from preliminary studies and based on the Cho et al. report [5]. The MWCNTs/TiO_2_ bi-layer coating was prepared in two steps. First, the electrophoretic suspension comprised of 0.1 g MWCNTs suspended in 40 mL of distilled water was prepared and the EPD process on the Ti13Nb13Zr substrate was proceeded. At this stage, preparation of the MWCNTs coating was finished. The MWCNTs coated samples were then subjected to EPD in suspension consisting of 0.15 g TiO_2_ dispersed in 50 mL of isopropanol and 0.5 mL of surfactant Polysorbate 20 (Tween 20, Sigma-Aldrich Sp. z.o.o, Poznan, Poland).

To prepare the MWCNTs_Cu dispersion coating, 0.1 g of MWCNTs, 0.005 g of nanoCu, and 0.5 mL of Polysorbate 20 were dispersed in 40 mL of distilled water and deposited using the EPD method on the Ti13Nb13Zr substrate.

### 2.3. Structure and Morphology

To study the surface topography, the optical microscope (VHX Keyence, Keyence International (Belgium) NV/SA, Mechelen, Belgium) was used. The average roughness index Sa values were estimated based on 512 lines made in the area of approximately 150 μm × 140 μm. 

The specimens’ surfaces and cross-sections were observed using a high-resolution scanning electron microscope (SEM) (JSM-7800F, JOEL, Tokyo, Japan) with a LED detector, at 5 kV acceleration voltage.

The chemical composition of the coatings was investigated by the X-ray energy dispersive spectrometer (EDS) (Octane Elite 25, EDAX Ametek, Berwyn, PA, USA).

### 2.4. Nanoindentation Studies

Nanoindentation tests were performed with the NanoTest™ Vantage (NanoTest™ Vantage, Micro Materials, Wrexham, Great Britain) using a Berkovich three-sided pyramidal diamond. The twenty-five measurements were carried out on each sample. The maximum applied force was 10 mN, the loading and unloading rate were set up at 20 s, and the dwell period at maximum load was 10 s. The distances between the subsequent indents were 20 μm. During the indent, the load–displacement curves were determined by the Oliver and Pharr method. Based on the load–penetration curves, surface hardness (H), reduced Young’s modulus (E_r_), plasticity index (PI), and elastic recovery index (EI) were calculated using the integrated software. Young’s modulus (E) parameter was calculated based on E_r_ value and the Poisson’s ratio (ν). The ν of 0.25 [67] was preconceived for the MWCNTs coating, 0.26 for TiO_2_ [68], and 0.352 for Cu [69]. Assuming the MWCNTs/TiO_2_ and MWCNTs_Cu coatings as unidirectional composite materials, the Halpin–Tsai (H-P) model was used to evaluate their ν, E [70], and H [71] values using the following relations:

(1) $$\nu =\nu_{m}V_{m}+\nu_{r}V_{r}$$

(2) $$E=E_{m}V_{m}+E_{r}V_{r}$$

(3) $$H=H_{m}V_{m}+H_{r}V_{r}$$

where V_r_, ν_r_, E_r_, H_r_ and V_m_, ν_m_, E_m_, H_m_ are volume fraction, Poisson’s ratio, Young’s modulus, and hardness of the reinforcement and matrix, respectively. The volume fraction was calculated according to [71] and assuming densities of 2.10 g/cm^3^ [72], 4.23 g/cm^3^ [73], and 8.94 g/cm^3^ [74] for MWCNTs, TiO_2_, and nanoCu, respectively. 

According to Equation (1), the ν values of 0.254 and 0.251 were assessed for the MWCNTs/TiO_2_ and MWCNTs_Cu coating, respectively.

## 3. Results and Discussion

### 3.1. Structure and Morphology

**Figure 1:**
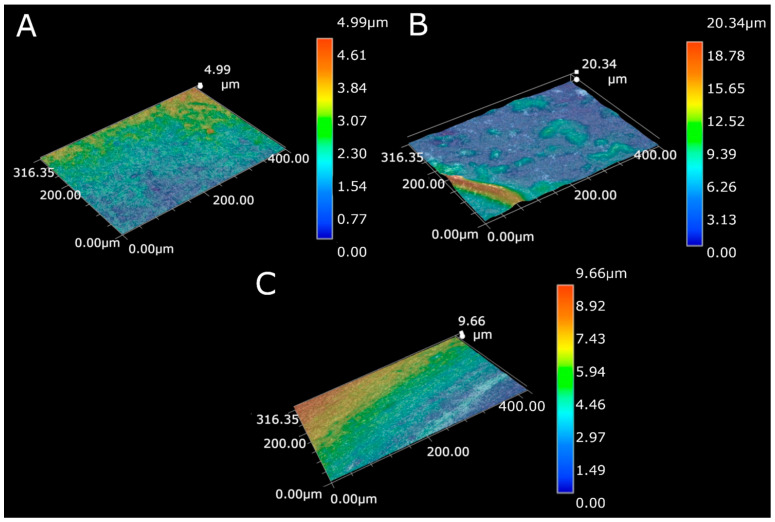
Surface topography for (**A**) the MWCNTs coating, (**B**) the MWCNTs/TiO_2_ coating, (**C**) the MWCNTs_Cu coating.

Figure 1 shows the topography of the MWCNTs coating, MWCNTs/TiO_2_ bi-layer coating, and MWCNTs_Cu dispersion coating. The coating surface roughness increased after composition modification with both metal and oxide particles, as proven by the results presented in Table 3 (the experimental error has been estimated as of <0.05 µm). A similar effect was shown previously in our earlier work [57] for MWCNTs coating with the addition of nanoCu particles. The observed effects are easy to explain. For bi-layer coating, small titania particles located on the surface form a rough surface. For dispersion coating, the geometries of MWCNTs and nanocopper particles are different, i.e., misfit occurs. The increase in hardness is not large and can be positive for biological applications as such roughness enhances the adhesion of cells.

**Figure 2:**
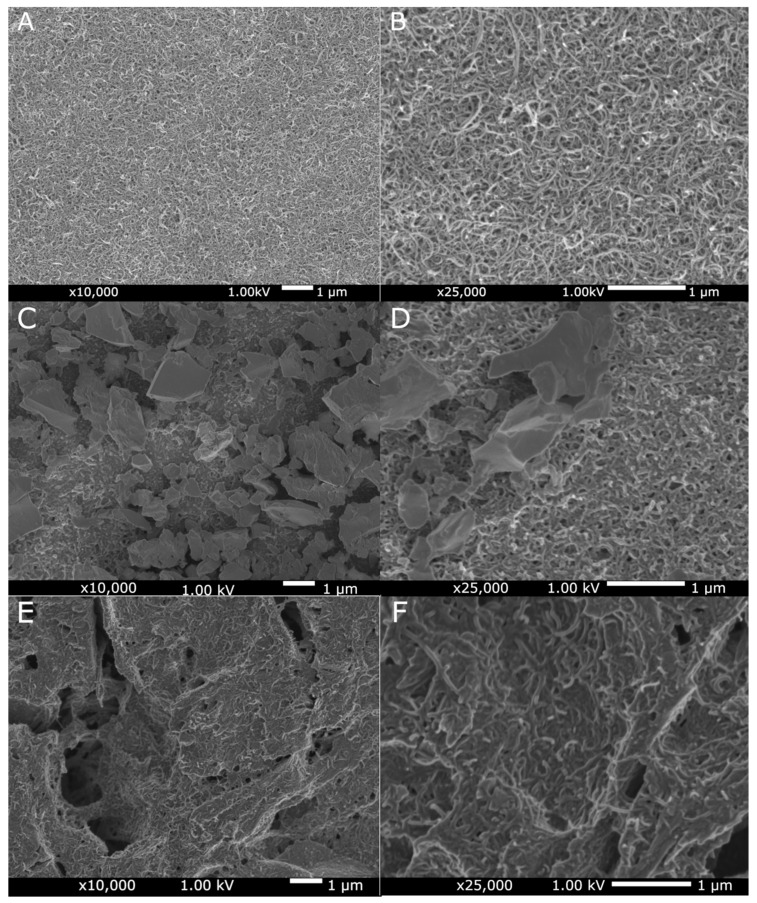
SEM surface topography of the MWCNTs coating (**A**,**B**), the MWCNTs/TiO2 bi-layer coating (**C**,**D**) and the MWCNTs_Cu dispersion coating (**E**,**F**) in different resolutions.

**Figure 3:**
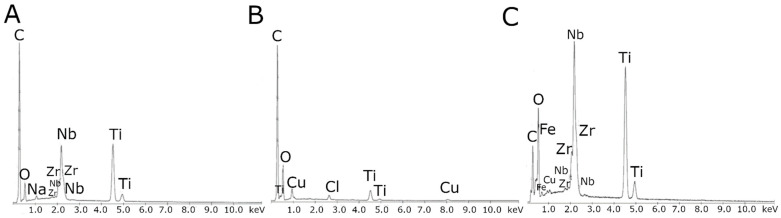
EDS spectra of the (**A**) MWCNTs reference coating, (**B**) MWCNTs/TiO_2_ bi-layer coating, (**C**) MWCNTs_Cu dispersion coating.

Figure 2 present SEM images of the MWCNTs sample, the MWCNTs/TiO_2_ bi-layer coating, and the MWCNTs_Cu dispersion coating deposited on the Ti13Nb13Zr alloy. Figure 2 demonstrates the uniform distribution of MWCNTs. Figure 2C,D illustrates a more diverse surface with visible agglomerates of many TiO_2_ particles with an average aggregates surface area of about 1.5 µm^2^. The surface topography of the MWCNTs_Cu coating (Figure 3E,F) is different: The MWCNTs coating is significantly cracked due to deposition of nanoCu particles, especially at the crossover and edge-contact locations of MWCNTs as previously observed [28,75,76]. Such a phenomenon can be attributed again to a significant difference in geometry (misfit) in both nanoforms.

**Figure 4:**
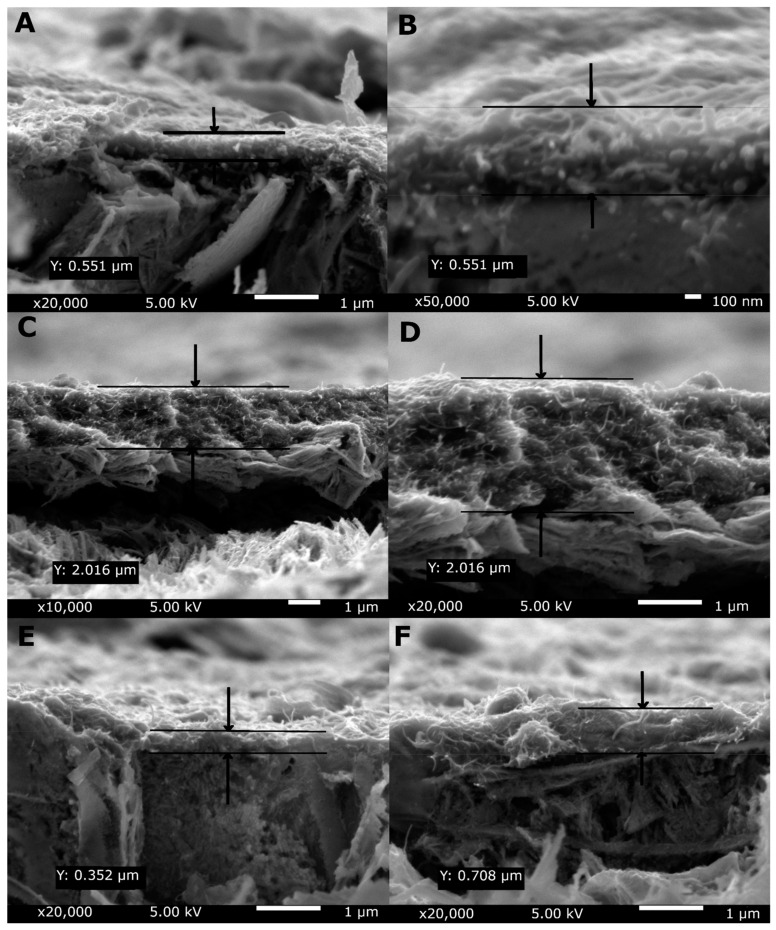
Cross-sectional SEM images of the MWCNTs reference coating (**A**,**B**), MWCNTs/TiO_2_ bi-layer coating (**C**,**D**) in different resolutions and MWCNTs_Cu dispersion coating (**E**,**F**) in different places, with an indicated coating thickness.

Figure 3 illustrates the EDS spectrum of the MWCNTs, the MWCNTs_Cu dispersion coating, and the MWCNTs/TiO_2_ bi-layer coating, confirming its formation by the presence of carbon, copper, titanium, and oxide peaks together with those from alloying elements (except the MWCNTs/TiO_2_ coating as a result of the largest thickness of examined coatings, which is shown in Figure 4), and iron, sodium, chlorine, and copper in MWCNTs/TiO_2_, likely as possible contaminants. 

Figure 4 shows the cross-sectional SEM images of the MWCNTs reference coating, the MWCNTs/TiO_2_ bi-layer coating, and the MWCNTs_Cu dispersion coating with an indicated coatings thickness (Y). As seen, the thickness of the coatings, shown in both images, extended roughly between 0.5 and 2 µm. The greatest values are observed for the MWCNTs/TiO_2_ coating which could be explained by the deposition method resulting in the bi-layer coating. What is important, all coatings possess the thickness sufficient to separate the substrate surface, with the greatest thickness of the MWCNTs/TiO_2_ coating being the most appropriate for application as sufficiently thick for retardation of possible penetration of liquids.

### 3.2. Nanoindentation Studies

**Figure 5:**
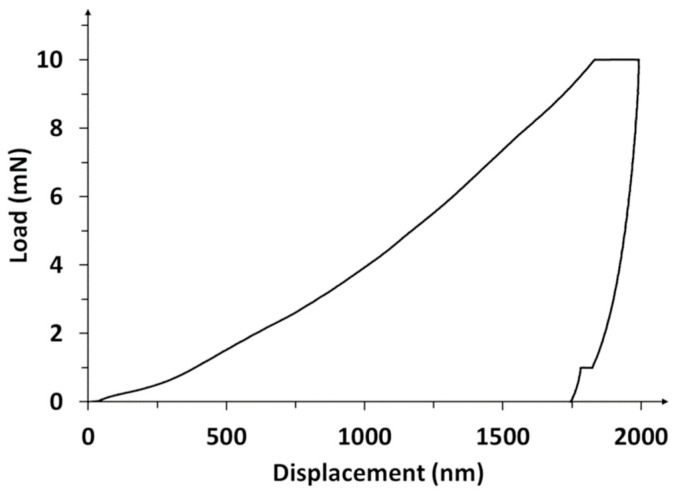
Nanoindentation load–displacement curve obtained for the MWCNTs/TiO_2_ coating.

The load–displacement hysteresis curve, as an example of the results of nanoindentation tests, is shown in Figure 5. Three stages of the nanoindentation test can be distinguished in the graph: raising the load until maximum value, a pause (to stabilize the probe at maximum depth), and offloading. Due to temperature drift, an irregularity in the form of the stage can be observed. The drift is adjusted at the end of nanoindentation.

The exact values of measured mechanical properties are listed in Table 4. The addition of both metal and oxide particles resulted in an increase in nanohardness over 100% and over 36%, respectively, with regard to the reference. The composite coatings demonstrate Young’s modulus lower than that of the reference coating and measured values are approximately close to that of bone, reported as of 18.6 GPa for cortical bone [63], but only 1 GPa for immature and 6 GPa for mature bone [77]. 

Such behavior, an inverse relationship between hardness and Young’s modulus, already anticipated by us [67], has been achieved. The decreasing Young’s modulus may be explained by several reasons as the most obvious strengthening of material under nanoindenter load by freshly created dislocations [78]. However, we believe that the main mechanism is related to the presence of local pores/voids in the microstructure of investigated composite coatings. As well-known, for homogenous composites, both hardness (strictly: strength) and Young’s modulus are additive values calculated based on values of mechanical properties and relative fractions of components. Table 4 shows the values of hardness and Young’s modulus for pure components (for MWCNTs twice, as isolated nanofilm and as a coating on Ti alloy). Taking into account the weight fractions in composite coatings, theoretical hardness, and Young’s modulus values were calculated (using Equations (2) and (3)).

Assuming that Young’s modulus values are also additive, porosity might be expressed as a ratio of real Young’s modulus to the calculated above value. However, such an approach is unjustified as it is no perfect composite. Zhang et al. tried to find a relation between porosity and mechanical properties (such as microhardness and Young’s modulus) and reported that, from the statistical trend, the coating porosity increases when microhardness decreases, the same as Young’s modulus decreases [82]. In literature, porosity phenomenon is related to a presence of empty spaces, named pores, and manifests itself as lower density and compactness of porous material than those of the same bulk material. Most methods evaluating the porosity of the coatings are based on image analysis, which is subjective [82,83]. On the other hand, coating porosity affects also the corrosion resistance of the coating’s substrate, as revealed in [83,84]. Besides, as Praveen et al. reported [85], the CNTs addition to zinc deposit reduces pore volume, and thus improves the corrosion resistance of CNT-Zn coatings. The decrease in Young’s modulus can be caused by pores which do not oppose the nanoindenter, but also by weak adhesion between MWCNTs and other nanoparticles. The physical image of occurring processes is different in solid materials and composites. Moreover, this composite coating has a specific structure: the main component, always placed on a metallic base, is a film composed of carbon nanotubes. If the ceramic layer is deposited on the previous MWCNTs layer, the randomly positioned nanotubes relatively easily bend off under a load of the penetrating nanoindenter and allow the layer comprised of titanium oxide nanoparticles also to deflect. Thus, the elastic elongation is easy and Young’s modulus is low. For the dispersion composite coating with nanocopper particles, the course of the process is similar even if, in this case, the nanocopper particles directly penetrate the “forest” of nanotubes not encountering any serious opposition, to some extent. 

The hardness, on the other side, has an understandable relation with the strengths of the components. The higher hardness values of layers comprising of titania or nanocopper can be explained by a specific nanohardness measurement which starts at the surface provided then that the minimal hardness is required to begin measurement (no test in the air might start). In such a case, in composite coatings, the hardness values which are means of several measurements may fulfill the relation between properties of composites, properties of each component, and fractions of individual components (Table 4). Differences between hardness values here measured and those assessed by the Halpin–Tsai model are simple to understand as the considered model has been prepared for composite materials in which there have been no empty spaces (pores, voids, cracks) and, on the other hand, a specific nature of isolated various nanoforms, bonded by presumably weak chemical and physical bonds. Despite that, these differences in hardness are not very high and, therefore, the obtained coatings may be assumed as of composite type, roughly approaching the properties of ideal composite solid. The applicable models for such composites which would take into account the inhomogeneous structure of a solid and adhesion forces between nanoparticles are, unfortunately, unknown at the moment. 

Even more exciting is a comparison of real and theoretical values for Young’s modulus. For both coatings, these values are lower than those predicted by the model, and again the 3D structure is useful to understand such behavior. For the bi-layer, the nanoindenter tip likely bends the titania layer and this motion is not substantially impeded by the MWCNTs inner layer with its long tubes, random positioning and significant free space between nanotubes. For MWCNTs_Cu, the mechanism of deformation is different and related likely more to the misfit between MWCNTs and nanocopper particles creating additional free spaces. Therefore, the difference between real and anticipated values is, in the last case, distinctly lower than for the bi-layer coating.

**Figure 6:**
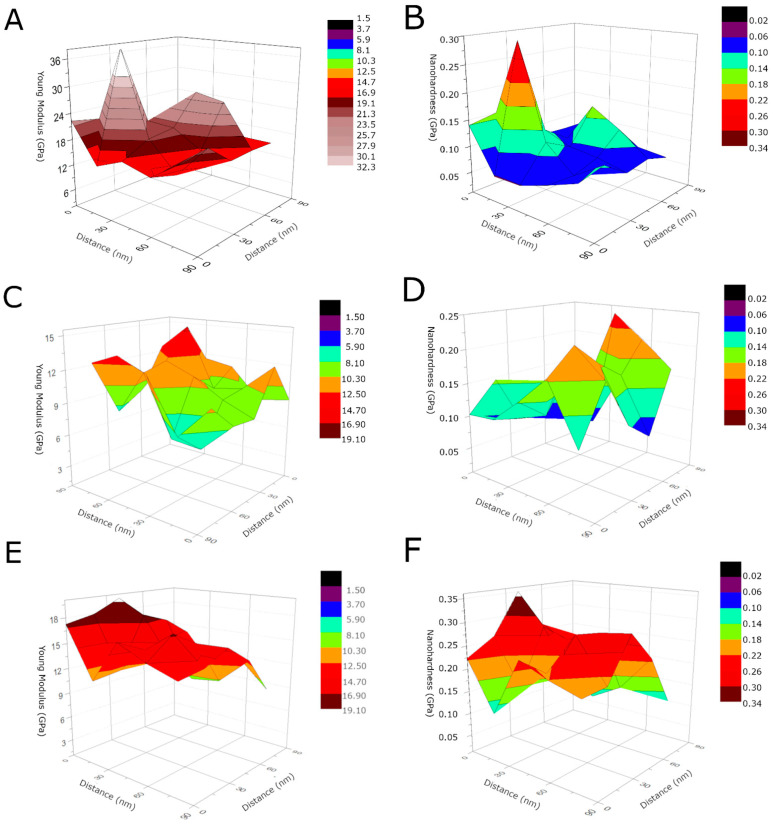
3D Young’s modulus and nanohardness distribution for the MWCNTs sample (**A**,**B**), the MWCNTs/TiO_2_ coating (**C**,**D**), the MWCNTs_Cu coating (**E**,**F**), respectively.

Figure 6 shows the 3D Young’s modulus and nanohardness distribution for the MWCNTs sample, the MWCNTs/TiO_2_ bi-layer coating, and the MWCNTs_Cu dispersion coating. As high fluctuations of both values can be observed depending on the specific measured area, these results are evidence that the coatings possess a highly non-uniform rough surface as already shown in topography tests and by great standard deviations in Table 5. The 3D-mechanical properties’ distributions demonstrate “elevations”, which result from the probe’s contact with the surface of the native material for the MWCNTs reference coating, the presence of TiO_2_ agglomerates for the MWCNTs/TiO_2_ bi-layer coating and the presence of nanoCu aggregates, or in the aftermath of the microcracks, the probe’s contact with the substrate of the MWCNTs_Cu dispersion coating. 

Generally, when hardness decreases, Young’s modulus decreases as well, and vice versa, as shown by several examples such as for Ti-based alloys with Ta and Fe additions [86], MWCNTs [87], La_2_O_3_-modified HfC-SiC coating for SiC-coated C/C composites [88], HAp-Ti-CNTs [4], YSZ-C-alumina [89], Ti-C-TiN coatings [90], MWCNTs/graphene Al nanocomposite [91], YSZ/Al_2_O_3_ coatings [92], MWCNTs/HAp [4,9,93], and our previous results [67], but also such simple relationships have not been observed in presence of TiO_2_ in BN sheets [94] and for Cu-Si composites [95]. Moreover, as perfectly shown in the review [96], nanoindentation measurements in multiphase solids are very complicated. For coatings, also the ratio of indenter displacement to coating thickness as it is important to know whether coating or substrate determines the indentation response. 

Among the problems that contribute to the difficulty in the application of indentation techniques to natural inhomogeneous materials is the presence of pores, microcracks, and voids [97]. In porous materials, mechanical properties usually decrease with increasing porosity as flexural strength and hardness in ZrB_2_-based composites [98], hardness in porous chromium carbide [99], Young’s modulus for plasma-sprayed YSZ coatings [100]. In [101], Young’s modulus and strength of single-phase TCP distinctly decrease with increasing porosity, for the first property over 50% at 20% porosity, but toughness was independent on porosity. On the other hand, for Cr_3_C_2_ ceramics, hardness increased at increasing pore size in the range 0.8 to 3.5 µm [99], but no explanation of this observation was proposed. Even for ZrO_2_-Y_2_O_3_ coatings, again the same relationship between Young’s modulus and hardness on porosity was observed, but only in some side regions so that this relation was sensitive to the morphology of the coating [102]. It is also to underline that hardness is related not only to elastic modulus but also to flexural strength, toughness, compression, and wear resistance in a complex way.

Two factors in multicomponent components determine mechanical properties. The first is the presence of tough particles, such as here, rutile nanograins and nanocopper particles (tough species in nanoform). The hard particles counteract the indenter penetration through the matrix due to the accumulation of dislocations in the deformed area. Another determinant is adhesion and bond strength between components [95]. We think that in tested composite coatings, the dislocation effect is negligible, hardness is determined by tough particles, but weak adhesion of components results in easy separation of nanoparticles and movement of the nanoindenter tip between them, utilizing the free spaces (intrinsic pores).

**Figure 7:**
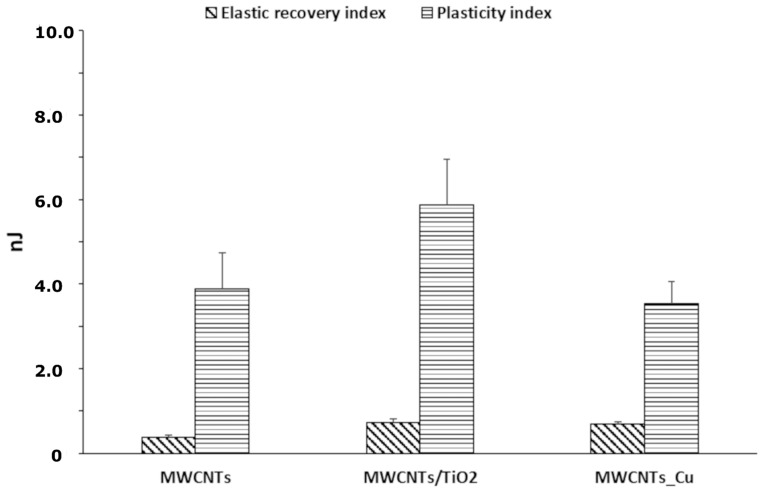
The diagram of EI and PI calculated for the MWCNTs coating, MWCNTs/TiO_2_ bi-layer, and MWCNTs_Cu deposition coating, examined under indentation load of 10 mN.

The mechanical behavior and the wear resistance of the examined coatings were further assessed from the nanoindentation results by evaluating elastic recovery index (EI), plasticity index (PI), ratio of the nanohardness to the reduced elastic modulus (H/Er) and yield pressure (H^2^/Er^3^). The EI represents the amount of energy released by the material under the influence of load. Whereas, the PI demonstrates the energy dissipated in the coating due to plastic deformation [86,87,103]. Figure 7 shows the EI and PI of the MWCNTs sample, the MWCNTs/TiO_2_ bi-layer and the MWCNTs_Cu dispersion coating deposited on the Ti13Nb13Zr alloy. The highest PI is observed for the MWCNTs/TiO_2_ coating, indicating its highest plastic deformation during the nanoindentation test, while the PI for MWCNTs_Cu is the lowest. Nevertheless, the EI of the nanoCu and TiO_2_-modified coatings were 2-fold improved in the contrary to the MWCNTs sample, which demonstrates the stiffening effect of the additives. 

**Figure 8:**
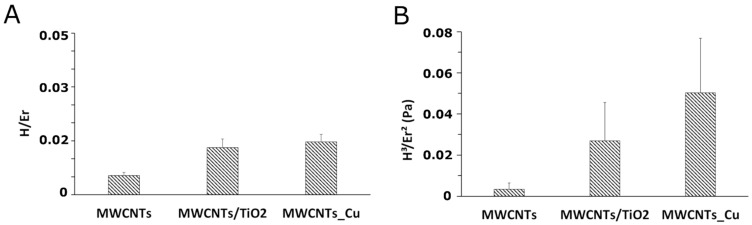
The diagrams for the H/Er (**A**) and the H^3^/Er^2^ parameter (**B**) calculated for the MWCNTs bi-layer and the MWCNTs_Cu dispersion coating, examined under indentation load of 10 mN.

Further crucial parameters are the ratio of the nanohardness to elastic modulus (H/Er), which shows the endurance capability of a surface coating, especially its ability to accommodate substrate deflections under load [103], and the yield pressure (H^3^/Er^2^), used to evaluate the resistance to plastic deformation of materials under nanoindenter tip load [56]. The H/Er and H^3^/Er^2^ parameters for the MWCNTs sample, the MWCNTs/TiO_2_ bi-layer, and the MWCNTs_Cu dispersion coating, examined under indentation load of 10 mN are shown in Figure 8. The addition to the MWCNTs coating-based Ti13Nb13Zr substrate, several nanoCu or TiO_2_ particles improves the wear resistance under surface deflections and the resistance to plastic deformation under applied load [86]. This means that the higher the H^3^/Er^2^ parameter is, the higher the fraction of plastic work during stress applying on coating surface should be. Conversely, the diagram showed in Figure 8 demonstrates the lowest PI for the MWCNTs_Cu dispersion coating, what indicates the release of energy dissipated in the coating under nanoindenter tip load and explains the appearance of the cracks shown in Figure 2.

These results are favorable for both composite coatings. These coatings are innovative and, being able to oppose some stresses during and after implantation surgery, are plausible for clinical practice. Moreover, they potentially may express some antibacterial efficiency, all of the tested components. However, the coating composed of two layers, inner MWCNTs and rutile outer layer, is sufficiently thick and does not contain Cu, that above certain limits of content might be toxic for a biological organism. Therefore, in the next step, we are going to verify, for both coatings, their biological properties, including their wettability, bioactivity of live cells, cytotoxicity, and antibacterial efficiency.

## 4. Conclusions

In this study, the influence of titanium dioxide and nanocopper additions on the mechanical properties of the Ti13Nb13Zr substrate covered with the multi-wall carbon nanotube-based nanocomposites was investigated. The mechanical properties of the coatings were evaluated using the nanoindentation technique and the following conclusions were drawn from the investigations:

(1) The results show Young’s modulus value tends to decrease with rising nanohardness, which is a positive effect resulting from the structure of composite coatings, i.e., a simultaneous presence of elastic carbon nanotubes and tough nanoparticles of copper or rutile.
(2) Both composite coatings demonstrate the mechanical properties better than the MWCNTs coating on Ti alloy. The additions of nanocopper or titanium dioxide to the MWCNTs coating-based Ti13Nb13Zr alloy substrate improve plastic and elastic recovery index, wear resistance to surface deflection, and to plastic deformation under applied load.
(3) Comparing composite coatings to each other, the dispersion coating with nanocopper has distinctly higher hardness, slightly higher yield pressure and Young’s modulus, comparable endurance capability and elasticity recovery index, and substantially lower plasticity index. On the other side, the bi-layer coating has the greatest thickness combined with a satisfactory ability to accommodate the substrate under applied load, and the highest plasticity index, which indicates its best resistance to plastic deformation.
(4) The yield pressure parameter is strictly related to the plasticity index, which shows the endurance of material to plastic deformation.
(5) The observed stiffening effect can be attributed to dislocation strengthening under a load of the nanoindenter tip in presence of tough and hard nanoparticles.
(6) The noticed decreasing Young’s modulus in both composite coatings compared to the MWCNTs coating may be explained by an appearance of porosity of coatings.

## Figures and Tables

**Table 1:** Chemical composition of the Ti13Nb13Zr alloy.

Element	Nb	Zr	Fe	C	H	O	S	Hf	Ti
wt. pct.	13.18	13.49	0.085	0.035	0.004	0.078	<0.001	0.055	rem.

**Table 2:** Parameters of synthesis of coatings.

Coating	Substrate	Deposited Materials	Content of Component in a Bath (wt. pct.)	EPD Time (min)	EPD Voltage (V)
MWCNTs	Ti13Nb13Zr	MWCNTs	0.25	0.5	20
MWCNTs/TiO_2_	Ti13Nb13Zr	(I) MWCNTs	0.25	0.5	20
		(II) TiO_2_	0.30	4	50
MWCNTs_Cu	Ti13Zr13Nb	MWCNTs + nanoCu	0.25 0.0125	4	50

**Table 3:** The surface roughness of the deposited coatings.

Sample	Roughness Sa (µm)
MWCNTs	0.34
MWCNTs/TiO_2_	0.65
MWCNTs_Cu	0.41

**Table 4:** A statement of weight fraction, real H, and real E parameters for pure components and MWCNTs composite coatings, compared with H and E parameters calculated based on H-P model for MWCNTs composite coatings.

Material	Weight Fraction of MWCNTs	Weight Fraction of TiO_2_	Weight Fraction of nanoCu	Calculated H (GPa)	Calculated E (GPa)	Real H (GPa)	Real E (GPa)
Ti13Zr13Nb alloy	0	0	0	-	-	3.760 [67]	83.32 [67]
MWCNTs	1	0	0	-	-	0.204 [79]	2.659 [79]
TiO_2_	0	1	0	-	-	1.000 [80]	68.00 [80]
nanoCu	0	0	1	-	-	1.200 [81]	104.20 [69]
MWCNTs coating	1	0	0	-	-	0.101	14.17
MWCNTs/TiO_2_ coating	0.4	0.6	0	0.485	37.15	0.137	7.69
MWCNTs_Cu coating	0.952	0	0.048	0.114	15.22	0.213	10.83

**Table 5:** Mechanical properties and maximum indent depth for the substrate and achieved coatings.

Sample	Nanohardness H (GPa)	Reduced Young’s Modulus Er (GPa)	Young’s Modulus E (GPa)	Maximum Indent Depth (µm)	Plasticity Index PI (nJ)	Elastic Recovery Index EI (nJ)
MWCNTs	0.101 ± 0.049	18.59 ± 5.66	14.17 ± 4.32	2.07 ± 0.35	3.88 ± 0.85	0.378 ± 0.056
MWCNTs/TiO_2_	0.137 ± 0.048	10.22 ± 2.33	7.69 ± 1.75	1.81 ± 0.33	5.87 ± 1.08	0.722 ± 0.084
MWCNTs_Cu	0.213 ± 0.061	14.28 ± 2.80	10.83 ± 2.12	1.43 ± 0.23	3.53 ± 0.53	0.688 ± 0.065

## Data Availability

Not applicable.
